# Causal Responsibility and Robust Causation

**DOI:** 10.3389/fpsyg.2020.01069

**Published:** 2020-05-27

**Authors:** Guy Grinfeld, David Lagnado, Tobias Gerstenberg, James F. Woodward, Marius Usher

**Affiliations:** ^1^School of Psychology, Tel Aviv University, Tel Aviv, Israel; ^2^Cognitive, Perceptual, and Brain Sciences Department, Experimental Psychology, University College London, London, United Kingdom; ^3^Stanford University, Stanford, CA, United States; ^4^Department of History and Philosophy of Science, University of Pittsburgh, Pittsburgh, PA, United States; ^5^Sagol School of Neuroscience, Tel Aviv University, Tel Aviv, Israel

**Keywords:** causality and responsibility, attributions of responsibility, robust causation, causal contingency and stability, epistemic perspective

## Abstract

How do people judge the degree of causal responsibility that an agent has for the outcomes of her actions? We show that a relatively unexplored factor – the robustness (or stability) of the causal chain linking the agent’s action and the outcome – influences judgments of causal responsibility of the agent. In three experiments, we vary robustness by manipulating the number of background circumstances under which the action causes the effect, and find that causal responsibility judgments increase with robustness. In the first experiment, the robustness manipulation also raises the probability of the effect given the action. Experiments 2 and 3 control for probability-raising, and show that robustness still affects judgments of causal responsibility. In particular, Experiment 3 introduces an Ellsberg type of scenario to manipulate robustness, while keeping the conditional probability and the skill deployed in the action fixed. Experiment 4, replicates the results of Experiment 3, while contrasting between judgments of causal strength and of causal responsibility. The results show that in all cases, the perceived degree of responsibility (but not of causal strength) increases with the robustness of the action-outcome causal chain.

## Introduction

The causal responsibility an agent has for the effects of her actions is thought to play a major role in the attribution of the agent’s legal, moral and even criminal responsibility (Hart and Honoré, 1959; Tadros, 2005; Moore, 2009; Lagnado and Gerstenberg, 2017; Usher, 2018). Indeed, causal responsibility is a necessary condition for the ascription of legal responsibility (Hart and Honoré, 1959; Tadros, 2005). Research in moral psychology has identified general cognitive processes such as causal and intentional attributions to explain patterns of responsibility judgments in both moral and non-moral domains (Cushman and Young, 2011; see also Spranca et al., 1991; Royzman and Baron, 2002; Mikahil, 2007; Waldmann and Dieterich, 2007; Lagnado and Channon, 2008; Baron and Ritov, 2009; Greene et al., 2009). For example, Cushman and Young (2011) show that action versus omission and means versus side-effect differences in moral judgments are mediated by their effects on non-moral representations of causal and intentional attributions.^1^ Similarly, Mikahil (2007) accounts for the means versus side-effect distinction in terms of action plans which specify generic rather than morally specific reasoning (see also Kleiman-Weiner et al., 2015). Furthermore, the reduced judgments of responsibility people typically make for agents who were forced to act or were manipulated by others (Sripada, 2012; Phillips and Shaw, 2015) are best accounted for by the manipulation “bypassing” the agent’s mental states (Murray and Lombrozo, 2017). To explain these results, Murray and Lombrozo (2017) relied on the notion of *counterfactual robustness*, which is central to our investigation and on which we elaborate below.

While causation is basic in all sciences, its proper understanding has been the subject of intensive recent development and debate in philosophy (Pearl, 2000; Woodward, 2003; Hitchcock, 2007; Halpern, 2016). A key distinction is between type and token level causation. The former applies to laws and generalizations (such as smoking causes cancer), while the latter applies to particular cases (John’s smoking caused his cancer). The concept of causal responsibility is typically grounded in the notion of token (or actual) causation. This means that to know that an agent is causally responsible for an effect, we need to know that she actually caused it (in that particular case). However, while the classical analysis of actual causation is based on a *necessity* counterfactual – if C had not occurred, then E would not have occurred (Hume, 1748; Lewis, 1973) – which is an all-or-none concept (the counterfactual is either true or false) – judgments of causal and legal responsibility are *graded*. For example, an agent generally bears more responsibility for an effect that is a direct outcome of her action, than for an effect that results at the end of a long causal chain (Brickman et al., 1975; Spellman, 1997; McClure et al., 2007; Lagnado and Channon, 2008; Hilton et al., 2010). This is aptly illustrated in the Regina v. Faulkner (1877) legal case, in which a lit match aboard a ship caused a cask of rum to ignite, causing the ship to burn and resulting in a large financial loss by Lloyd’s insurance, leading to the suicide of a (financially ruined) insurance executive.^2^

Experimental studies of actual causation have also shown that judgments of causal strength and of causal responsibility vary with the typicality of the cause and the background conditions (Hilton and Slugoski, 1986; Kominsky et al., 2015; Samland and Waldmann, 2016; Icard et al., 2017; Gerstenberg et al., 2018) and also with the degree of causal redundancy (Gerstenberg and Lagnado, 2010; Zultan et al., 2012; Lagnado et al., 2013; Gerstenberg et al., 2015; Koskuba et al., 2018). For example, Kominsky et al. (2015) show that people are more likely to endorse an event (Alex’s coin-flip coming up heads) as causally responsible for another event (Alex wins the game), when the contingency between the two is high (Alex wins if both the coin comes up heads and the sum of two dice being thrown is *greater than 2*) than when the contingency between the two events is low (Alex wins if both the coin comes up heads and the sum of two dice being thrown is *greater than* 11). Also, when there is causal redundancy, for example, when the action of several agents overdetermines an outcome (e.g., two marksmen are shooting a person; Lagnado et al., 2013), the responsibility of each agent is reduced the more overdetermined the outcome is.

The interventionist framework (Pearl, 2000; Woodward, 2003) provides a general framework for understanding causal claims at both type and token level (we focus on the latter here). On this approach, X causes Y if some potential manipulation of X would lead to a change in Y, under suitable background conditions (for full details see Woodward, 2003). Theoretical work within this framework has highlighted two ways to extend the classical analysis of causality to provide room for degrees of causal responsibility. The first involves a refinement of the *necessity* condition (Chockler and Halpern, 2004; Halpern and Hitchcock, 2015), which suggests that necessity is to be tested not only on counterfactuals that negate the cause and keep all other co-factors constant, but also on counterfactuals that can vary some of these co-factors. For example, in situations of redundant causation (e.g., two marksmen shooting a person), neither of the agents’ actions is necessary for the outcome (either shot on its own was sufficient to kill the victim, so the victim would still have died, even if one of the marksmen hadn’t shot). However, if we allow for a more flexible type of counterfactual test, where one marksman’s action is assessed under the contingency where the other marksman does not shoot, then both marksmen can be counted as causes of the victim’s death. Moreover, this extended counterfactual account fits with empirical data on graded causal judgments (Zultan et al., 2012; Lagnado et al., 2013).

The second way to introduce gradations of causal judgments involves a complementary causal condition: *robust sufficiency* (Lewis, 1973; Pearl, 1999; Woodward, 2006; Lombrozo, 2010; Hitchcock, 2012; Kominsky et al., 2015; Icard et al., 2017; Usher, 2018; Vasilyeva et al., 2018). Focusing on the simplest case, where we have one putative cause X of an effect Y, and a set of background circumstances B: X is robustly sufficient for Y if, given that X occurs, Y would still occur, even under various changes to the background circumstances. In contrast, the sufficiency of X for Y is non-robust (or highly *sensitive*) if, given X, Y would only occur under a very specific (narrow) set of background circumstances. Thus, we have a spectrum of degrees of robustness according to the range of background circumstances under which X would remain sufficient (i.e., be pivotal) for Y. This notion of robustness can be generalized to more complicated situations involving multiple causal factors (see Gerstenberg et al., 2015; Kominsky et al., 2015), and also to non-deterministic contexts where causes merely raise the probability of their effects (Hitchcock, 2017). Applied to the case of Regina vs. Faulkner, the causal chain from the lit match to the suicide of the insurance executive seems non-robust (and highly sensitive): it held only under this very specific set of background circumstances, and would have failed if only one of these factors had been different (see also Halpern and Hitchcock, 2015). Robust actions, on the other hand, are thought to involve some degree of stability (low sensitivity) on the impact of background circumstances that are external to the action itself (Woodward, 2006).

### Robust Sufficiency Versus Probability Raising

An alternative framework for quantifying causal strength employs the notion of *probability-raising* (Suppes, 1970; Cheng and Novick, 1992; Spellman, 1997; Fitelson and Hitchcock, 2011), where the strength of the relation between cause and effect corresponds to the degree to which the cause raises the probability of the effect (holding all else equal). This account usually focuses on type-level causal relations (Cheng, 1997), but has been extended to actual causation (Spellman, 1997; Cheng and Novick, 2005; Stephan and Waldmann, 2018). Although the framework suffers from notorious difficulties in distinguishing correlation from causation (Cartwright, 1989; Woodward, 2003; Pearl, 2009), it can be revamped to give a potential measure of causal strength (e.g., probability-raising through intervention).

While robust causes will often raise the probability of their effects more than non-robust causes, robustness and probability-raising are potentially distinguishable. For example, suppose that a doctor considers two possible drugs to treat a difficult medical condition that has a 20% chance of recovery if untreated. Drug X has a success rate of 60% in two possible background conditions (B1 and B2, whose presence is difficult to establish), while drug-Y has a 100% recovery in B1, but only 20% in B2. Overall, if we assume that B1 and B2 are equally probable, then both drugs yield the same recovery rate of (60% + 60%)/2 = (80% + 20%)/2 = 60%.^3^ However, Drug X is more robust, because the relation between X and recovery holds under a greater number of background conditions, (B1 and B2 for X versus only B1 for Y). We will exploit this kind of example to de-confound robustness and probability-raising in our two latest experiments (3 and 4) that focus on the causal-responsibility of an agent for the effects of her actions, using a design similar to one recently employed by Vasilyeva et al. (2018), in the context of causal generalizations and causal explanations. Note that defining robust-sufficiency in terms of the number of background circumstances, rather than in terms of probability raising, has two important advantages. First, an agent may know of different background circumstances which moderate the relationship between the cause and the effect, but not have information about the probabilities with which those background circumstances occur (or about the probabilities of the effect conditional on the cause in those circumstances) and, hence may not have the information to make reliable judgments of probability raising. In such cases, the agent may still be guided by an estimate of the number of different circumstances in which the cause leads to the effect — that is, the robustness of the cause/effect relationship. Second, there are theoretical reasons to prefer causal relations that are invariant in various ways, in particular invariant to changes in background conditions (Cheng, 1997; Woodward, 2003).^4^ We will return to the distinction between robustness and probability raising in the Discussion.

### Judgments of Causation vs. Judgments of Responsibility

Our interest here is in judgments of causal responsibility that agents have for the outcomes of their actions, which are an essential component of the type of responsibility that is involved in judgments of praise and blame. While it is beyond the scope of our paper to offer (or test) a full theory of praise/blame (Malle et al., 2014; Halpern and Kleiman-Weiner, 2018), we note that a common assumption is that there are two components in credit/blame attribution: (i) an intentional one (the agent needs to have intended and foreseen the outcome of her action and the intention must not be the outcome of manipulation by another agent; Lagnado and Channon, 2008; Sripada, 2012; Phillips and Shaw, 2015), and (ii) a causal one: the agent must be causally responsible for the outcome in virtue of an action she did (or failed to do). In this study, we will only focus on the second component, by keeping the intention present and fixed. In that sense, our judgment of interest, *causal-responsibility*, is similar to judgments of *causal strength* (but note that we focus on judging the responsibility of agents for an outcome of their actions). Probing causal strength typically asks “to what extent X caused Y,” while probing causal responsibility asks “how responsible is X for Y” (Sarin et al., 2017). Indeed, most studies in the field have probed participants with either of these measures with parallel effects (Murray and Lombrozo, 2017; Sarin et al., 2017). Moreover, Sytsma et al. (2012) have proposed that “the ordinary concept of causation, at least as applied to agents, is an inherently normative concept: Causal attributions are typically used to indicate something more akin to who is responsible for a given outcome than who caused the outcome in the descriptive sense of the term used by philosophers” (p. 815).

There are reasons, however, to expect that under certain conditions, causal and responsibility judgments may diverge, even when the agent’s intention is held constant (Chockler and Halpern, 2004). Consider, for example, a doctor that administers drug X (60% recovery in both B1 and B2; robust to background conditions) or Y (100% recovery in B1 and 20% in B2; sensitive to background conditions), in the example above, and assume that the background circumstance B1 takes place (and B2 does not). In such a case, we believe that there is a good reason to expect a dissociation. While the causal strength between the doctor’s action and the patient’s recovery is likely to be higher in the case of drug Y (which in the actual circumstance, corresponding to B1, increases the probability of the effect by 80%, compared with an increase of only 40% for drug X), the causal responsibility attributed to the agent for the same event, is likely to be higher for substance X, as X is more robust, and thus the outcome is less sensitive to external circumstances. This distinction is consistent with a recent theory of actual causation proposed by Chockler and Halpern (2004), who distinguish between judgments of causality, responsibility and blame. For example, they consider the case of a person being shot by a firing squad of 10 marksmen, only one of whom has a live bullet. According to Chockler and Halpern, while only the marksman with the live bullet caused the death of the person, the *blame* is divided between all 10 marksmen, reflecting the *epistemic* uncertainty of the marksmen agents. Here, we propose that judgments of causal responsibility elicit the same epistemic perspective change as those of blame. While this differs from the distinction made by Chockler and Halpern, we believe that in the presence of the intentional component^5^, the distinction between attributions of blame/praise and causal responsibility is more subtle and thus is beyond the scope of the present work. For that reason, we group these two aspects (causal-responsibility and credit/blame) together in this study.

There are two recent studies that are relevant, in particular, to our present work. The former examined judgments of credit and blame, which were shown to depend on two factors: (i) the degree of causal redundancy (or *pivotality*) and (ii) the amount of skill that people infer the agent to possess (Gerstenberg et al., 2018). People attribute more credit to an agent when their action has a higher contingency to the outcome, indicative of skill (rather than luck), and when their action is pivotal for bringing about the outcome. In another recent study, Vasilyeva et al. (2018) reported an effect of stability on judgments of causal generalizations and causal explanations for causal contingencies. In their study, participants made judgments under conditions of uncertainty about the factors that determine the causal contingency. Here, we aim to contrast judgments of causal responsibility and of causal strength for agents that bring about an effect in a robust vs. non-robust manner. Moreover, we will focus on situations in which the agents, but not the participants, face epistemic uncertainty. This allows us to test the impact of robustness under situations with minimum epistemic uncertainty (from the perspective of the judging participant), as well as tease apart robustness, causal strength, and causal responsibility (of agents for outcomes of their actions). We defer a more detailed discussion of the differences between our study and that of Vasilyeva et al. (2018) to the General Discussion.

### Overview of the Paper

This paper aims to empirically test the impact of robustness on judgments of causal responsibility in human agency. To do so, in all our experiments, we probe the extent to which a human agent is judged to be causally responsible for the intended outcome that resulted from her action. In Experiment 1 (which probes both judgments of causal responsibility of causal strength), we manipulate robustness in terms of the number of background conditions under which the action would bring about the effect^6^, and assess judgments of both causal responsibility and causal strength. However, as noted, robustness is often correlated with probability-raising, and Experiment 1 does not discriminate between these two factors. Thus, Experiments 2–4 probe causal responsibility and manipulate robustness while holding probability-raising constant. Experiment 3 teases apart the effects of robustness and skill on causal responsibility (Gerstenberg et al., 2018), by holding the agents’ skill level constant, while varying the robustness of their actions. Finally, in Experiment 4, we replicate Experiment 3 and extend the results to cases of failure, contrasting judgments of causal responsibility and of causal strength.

## Experiment 1

In this experiment, we probe robustness by manipulating the number of background conditions under which an action is likely to result in a positive outcome. To do so, we designed a game in which an agent throws a dart at a target to determine how many dice will be rolled (one, two, or three) by a computer, with the player winning the game if the sum total on the dice is six or greater. The number of dice rolled (one, two, or three) depends on the agent’s dart throw in the following way: if the dart lands in one of the two inner circles (rings 5–6) three dice are rolled, if it lands in one of the two middle circles (3–4), two dice are rolled, and finally, if it lands in one of the two outer circles (1–2), one die is rolled. While under all three contingencies a win is possible, the degree of robustness increases with the number of dice rolled.^7^ For the single die roll, there is only one configuration (a six) resulting in a win. When two dice are rolled, there are several more configurations that result in a win (e.g., 6,1; 5,1; 4,2; etc.; 26 out of 36 possible outcomes), and for three dice there are even more possible configurations (e.g., 2,2,2; 1,4,5; etc.; 206 out of 216 possible outcomes). In other words, the action (the dart throw) can cause the desired outcome (winning) under more, or less, background conditions (dice rolls). Observers in the experiment watched an animation in which an agent first throws a dart which lands on one of the rings (1–6) of the dartboard. After that, the corresponding number of dice were “randomly” rolled, the outcome of the roll revealed, and winning or losing declared. Half of the participants were asked to evaluate the degree of responsibility of the agent for the win/loss. The other half was asked to evaluate the causal strength between the agent’s throw and the win/loss. In this experiment, since robustness and probability raising co-vary, while the agent’s epistemic perspective is held constant, we expect attributions of causal responsibility and causal strength to show similar patterns (cf. Vasilyeva et al., 2018). In Experiment 4, we identify a context in which the two measures dissociate.

We anticipated three possible patterns of results: (i) judgments of responsibility/causal strength will not be affected by the dart-ring outcome, implying that they are only sensitive to the outcome (success or failure), (ii) judgments will increase monotonically depending on which ring (1–6) the dart landed in, implying that they are mainly determined by the agent’s perceived skill, (iii) judgments will increase in two steps, from ring 2 to 3, and from ring 4 to 5, implying that they are determined by the robustness of the dart throw (action)/game-win (effect) contingency to background conditions (outcomes of the dice rolls).

### Method

#### Participants

One hundred and two participants (46 females, 56 males; mean age = 33.7, *SD* = 9.7) took part in the experiment. Fifty of whom rated how much the agent’s throw was a cause for winning or losing. The other 52 participants rated the agent’s responsibility for the result. All participants were recruited via Amazon Mechanical Turk and received 1$ for participating.

#### Materials

**FIGURE 1:**
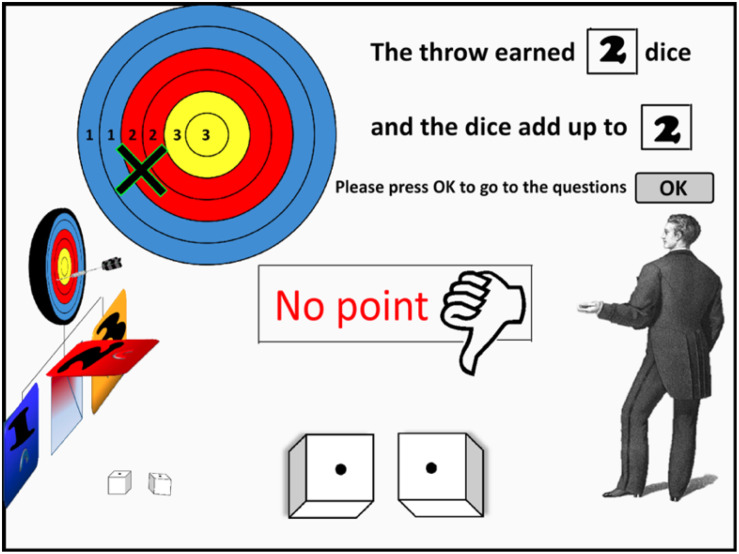
A screen-shot from the animation that participants watched in Experiment 1. The agent (on the right) throws the dart toward the board. The board is colored to illustrate the number of dice rolls earned. In this case, the arrow landed on Ring 3 (counting from the outside in), earning two dice. Each die landed on a 1, thus no point was won (a sum total for the dice of at least 6 was required to score a point).

Twenty video clips were created (see Figure 1 for illustration), which varied in terms of the ring in which the dart lands (1–6), the associated number of dice rolled (1–3), and the total score of the dice (2–12). The profiles of each of the 20 clips are shown in Table 1. Note that a total score of 6 or larger corresponds to a win, and scores lower than 6 to a loss. Here, we focus on the responsibility for success, but we included cases of failure in order to balance success and failures to make the game credible. We also constructed the set so that the total score of 6 (the critical value for success) appears for all possible ring numbers and number of dice thrown. This allows for a straightforward comparison of the influence of these variables on people’s ratings. When more than one dice was thrown, we included in addition winning cases in which the total scores were 9 or 12 (# of dice = 2, and # of dice = 3, respectively).

**TABLE 1:** Profiles of the 20 clips used in Experiment 1.

Clip	1	2	3	4	5	6	7	8	9	10	11	12	13	14	15	16	17	18	19	20
Ring	6	6	6	5	5	5	4	4	4	4	3	3	3	3	2	2	2	1	1	1
N. dice	3	3	3	3	3	3	2	2	2	2	2	2	2	2	1	1	1	1	1	1
Score	5	6	12	6	12	5	2	6	9	5	9	5	2	6	5	2	6	2	6	5
Result	L	W	W	W	W	L	L	W	W	L	W	L	L	W	L	L	W	L	W	L

*Ring = ring that the dart landed in (1 = most outer ring, 6 = center ring), dice = number of dice rolled, score = what sum the dice added up to, result = loss (l) or win (w). Scores of 6 or higher resulted in a win.*

#### Procedure

After having received instructions, each participant watched the 20 clips in randomized order. For each clip, participants watched a video showing a player throwing a dart toward the board, and landing on one of the six rings (1–6). Depending on the result of the dart throw, a number of dice (one, two or three) were rolled, and the total on the dice was displayed, along with notification of a win or a loss of a point (see Figure 1). Participants were then asked to enter their evaluation of responsibility (“to what extent is the player responsible for wining/losing of this point”) or of causal strength (“to what extent did the player cause the win/loss of this point”), by using a slider on a rating scale with endpoints labeled from ‘0 = not at all responsible (not at all the cause)’ to ‘10 = completely responsible (completely the cause).’

#### Analysis

Our main focus is on the win data, and in particular, when the total dice-score was equal to six, as this was the only winning outcome in all dice conditions. We thus carried out an ANOVA on the impact of the number of dice thrown (1, 2, or 3), of the ring number (odd vs. even), and of the type of judgment (responsibility vs. causal strength). Note that the odd/even categorical variable of ring number, contrasts between ring values (1, 3, 5) vs. (2, 4, 6), and thus tested if ring value matters once we control for the number of dice thrown. For completeness, we also ran a linear mixed-effects model on all success trials (where the total score is equal to or greater than 6), in which we predicted the causation and responsibility ratings from three variables: number of dice thrown, ring number (odd vs. even), and total score, as well as a participant dependent intercept. Finally, we ran exactly the same analyses for the cases of failures. Here, we replaced cases in which total dice score was 6 with those in which it was 5 in the ANOVA, and we also a linear mixed-effects model all failure trials with number of dice thrown, ring number and total score as predictors.

### Results

**FIGURE 2:**
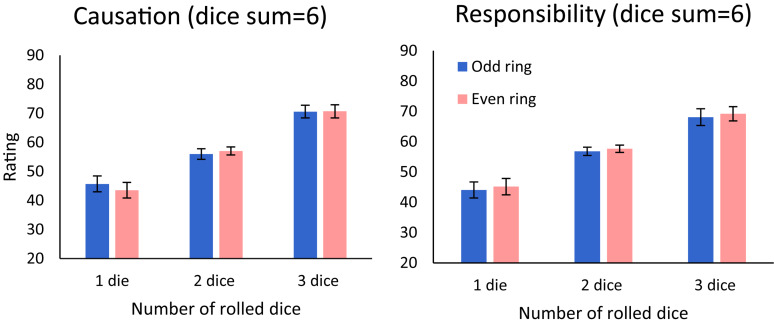
Evaluations of causal strength (left) and of responsibility (right) as a function of the number of dice rolled and of the ring the dart hit: odd (blue) vs. even (pink). Error bars indicate a within-subject ±1 standard error of the mean (Cousineau, 2005).

We start with the causal strength and causal responsibility judgments that are ascribed to the dart-throw agent for wins (total score ≥ 6). To illustrate the results, we plot in Figure 2 the mean ratings (for the total-score value of 6, which is the only win-condition, that appears in combination with all ring-values) for judgments of causal strength (left panel), and for responsibility (right panel), as a function of number of dice and ring (odd/even).

To test the impact of number of dice and ring-parity, we carried out a 3 × 2 × 2 ANOVA with number of dice rolled (one/two/three) and the ring-parity (odd vs. even) as within-subject factors, and judgment type (causation vs. responsibility) as a between-subject factor. The results showed a significant main effect for the number of dice rolled, *F*(2,200) = 55.21, *p* < 0.001, $\eta_{\text{p}}^{2}$= 0.356. *Post hoc* Bonferroni tests show that ratings for hitting the inner rings (three dice) were significantly higher than for the middle rings [two dice; *t*(100) = 7.57, *p* < 0.001, *d*= 0.750], which were significantly higher than outer rings [one dice; *t*(100) = 6.01, *p* < 0.001, *d* = 0.596]. No other main effects or interactions were significant (see Supplementary A for a report of the non-significant results).

This result was confirmed by a linear mixed effects model with random intercepts for each participant on all success outcomes trials, which showed that only the number of dice thrown was a significant predictor of responsibility judgments [*b* = 13.7, *t*(465) = 4.35, *p* < 0.001]. The regression coefficients for the ring [*b* = 1.2, *t*(465) = 0.82, *p* = 0.414] and the score [*b* = 0.5, *t*(465) = 1.41, *p* = 0.157] variables were not significant (see Supplementary A for the model predictions). We obtained similar results for the causation judgments. The regression coefficient was only significant for the number of dice [*b* = 13.8, *t*(447) = 4.287, *p* < 0.001]. The coefficients of the ring [*b* = 0.5, *t*(447) = 0.31, *p* = 0.754] and the total score [*b* = −0.2, *t*(447) = 0.60, *p* = 0.548] variables were not significant.

Finally, the results for the cases of failure, reflect those for the wins (see Supplementary A and Supplementary Figure S1). We find that the only variable that affects either judgment is the number of dice rolled – the variable that controls the success rate. The more dice were rolled (as a result of a better dart-throw), the lower was the ascription of causal responsibility to the agent for an eventual failure, and the lower was the extent to which people thought the agent caused the failure (see Supplementary A for full report of statistical tests).

### Discussion

As in previous studies (Gerstenberg et al., 2018), participants judged agents whose actions yielded higher success rates to be more responsible in case of success, but less responsible in cases of failure. The exact same pattern is shown for judgments of causal strength. Both the participants’ responsibility and causation judgments support the robust-causation hypothesis rather than the skill-only hypothesis. We reasoned that an agent’s variation in skill would correspond to more localized dart throw gradients (around the dart-board center, i.e., the 6-ring), and thus would result in more throws ending in even (2, 4, 6) compared with the odd (1, 3, 5) rings^8^. There was no such difference in either the causal strength nor the causal responsibility judgments. On the other hand, the robust causation hypothesis correctly predicts that people’s judgments of causation and responsibility are a function of the number of dice rolled, and not simply the closeness of the dart to the center of the board. The total score (which involves the luck of the dice throw) also did not affect the judgments either (as long as it was at least 6), when holding the number of dice constant, indicating that judgments were affected by the agent’s action, rather than by resultant luck (Nagel, 1979; Gerstenberg et al., 2010).

Nevertheless, one might argue that there is a simpler framework that can account for this pattern of results, which is independent of the notion of robustness. The idea is that as we increase the number of dice being rolled, we also increase the success probability, and an increased probability of success may lead to increased judgments of causal responsibility and causal strength (Suppes, 1970; Cheng and Novick, 1992; Spellman, 1997). Note, however, that probability-raising and robustness are not independent, and that one very typical way in which one increases the success probability of an action is by making it more robust. However, it is possible to tease apart robustness and probability-raising empirically. In the following experiments, we test the robustness hypothesis while keeping the probability of success fixed across conditions. To do so, we will contrast two types of actions whose success rate is the same, but which vary in their robustness. In Experiment 2, we use an animated soccer scenario, while in Experiments 3 and 4, we use a vignette based on an analog of Ellsberg’s ambiguity paradox.

## Experiment 2

In this experiment, we hold probability-raising constant and manipulate robustness in a more naturalistic setting – a soccer set-up based on video animations. Since we obtained in the previous experiment parallel results with the causal-strength and the responsibility measures, and since our main interest is the responsibility that agents have for the effects of their action, we explicitly probed here the responsibility and praiseworthiness of the agent. Participants view an animation with soccer players taking free-kicks, and with three defenders that form a (slightly moving) defensive wall in front of the goal (see *demo* link in the Materials section). The soccer players vary on two orthogonal factors – the probability of scoring a goal and the execution strategy (both manipulations were carried out within participants). In order to establish generality, we carried out two versions of this experiment, in which we manipulated the success probability by experience (Experiment 2a) and by description (Experiments 2b and 2c)^9^. There were two strategies: (i) The non-robust strategy of shooting directly into the wall (this may result in a goal, if the defenders happen to accidentally move out of the way). (ii) The robust strategy of shooting in a curved trajectory around the wall (if successful, this strategy is robust to the location of the defenders). Since a good execution based on the second strategy might be perceived as more difficult to achieve, we made it explicit that the probability of scoring a goal (for the robust and non-robust players) were identical. Participants evaluated the responsibility of four players (2 × 2 design) in scoring a specific goal in a free-kick for their team in a decisive match.

As predicted by both robustness and the probability-raising theory, we expected that participants would rate higher the responsibility of the players who have a higher scoring record, compared with players with a low scoring record whose goals may be perceived as “lucky” (cf. Johnson and Rips, 2015; Gerstenberg et al., 2018). As predicted by the robustness hypothesis, we also expected that responsibility judgments would be higher for players who bend their shots around the wall, even when the success rate is equated. Players who successfully bend their shot scored their goal in a way that is not dependent on the particular background circumstances (and thus are less “lucky”).

### Method

#### Participants

Twenty-two participants (4 female, 18 males; mean age = 28.67, *SD* = 8.45) were tested in Experiment 2a – the *experience*-condition. These participants were either Tel Aviv University students (16 participants) that received 15 min credits for participating in the lab, or volunteers (6 participants) who ran the experiment on remote computers via a link to the same Qualtrics site. Twenty participants (1 female, 19 males; mean age = 33.8, *SD* = 12.8) were tested in Experiment 2b (*description*-condition in which the success rate of each player was verbally stated). These participants were recruited via “Hamidgam project” (the Israeli equivalent of Amazon Mechanical Turk) and received a payment that was equivalent to 0.41$ for participating. Three additional participants were excluded because they reported internet connectivity problems (2), or because they didn’t watch or play soccer at least once a year (1). In Experiment 2c (replication of Experiment 2b), we tested 46 participants (46 males, mean age = 29.09, *SD* = 4.47) via “i-Panel.”^10^ These participants received a payment that was equivalent to 0.27$. All participants across all three studies reported watching or playing football at least once a year.

#### Materials and Procedure

Participants took part in the lab or remotely, using their computer. After a short instruction, six sample free kicks for each player (experience condition) or a table with the player’s success rates and kick-style (description condition; see Supplementary B) were shown. Video-clips were shown and ratings were made using the Qualtrics platform. The video clips may be accessed here: https://github.com/guygrinfeld/Responsibility-and-Robust-Causation-Experiments/tree/master/videos-sample.

**FIGURE 3:**
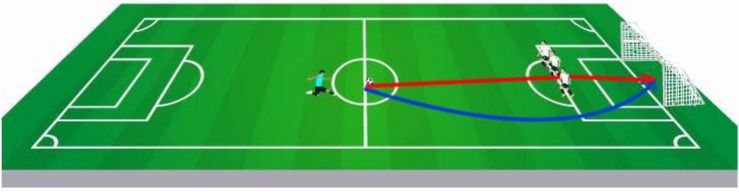
Illustration of the robust (curved, blue line) and non-robust (straight to the wall, red line) free kicks. In the animation, the defenders are moving, such that their locations are difficult to predict (see demo: https://github.com/guygrinfeld/Responsibility-and-Robust-Causation-Experiments/tree/master/videos-sample).

After watching each player (or reading information about each player), a video-clip of a successful free-kick was shown (see Figure 3) and subjects were asked to evaluate: “How responsible and praiseworthy is the player for this goal?” on a scale from 0 (not responsible at all) to 100 (has full responsibility). Four players were presented and rated sequentially, according to the same procedure. Each player had a different color shirt to help differentiate the players. The four players had the following characteristics:

(1) Robust player (curved trajectory), low success rate: In Experiment 2a, this player shoots the ball in a curved trajectory and succeeds in two out of six free-kicks (i.e., 1/3 success rate). Successful free-kicks were second and fourth. In the description condition, the success rate was stated as 30% in Experiment 2b and as 1/3 in Experiment 2c.
(2) Robust player (curved trajectory), high success rate: This player scores in four out of six free-kicks (i.e., 2/3 success rate). Successful free-kicks were first, third, fifth and sixth. In Experiment 2b, the success rate was stated as 60% and in Experiment 2c as 2/3.
(3) Non-robust player (straight trajectory), low success rate: In Experiment 2a, this player shoots the ball straight at the wall and succeeds in two out of six free-kicks (1/3 success rate). Successful free-kicks were second and fourth. In Experiment 2b, the success rate was stated as 30% and in Experiment 2c as 1/3.
(4) Non-robust player (straight trajectory), high success rate: This player scores four out of six free-kicks (2/3 success rate). Successful free-kicks were first, third, fifth, and sixth. The success rate was stated as 60% in Experiment 2b and as 2/3 in Experiment 2c.

The order of four players was randomly assigned to each participant.

### Results

**FIGURE 4:**
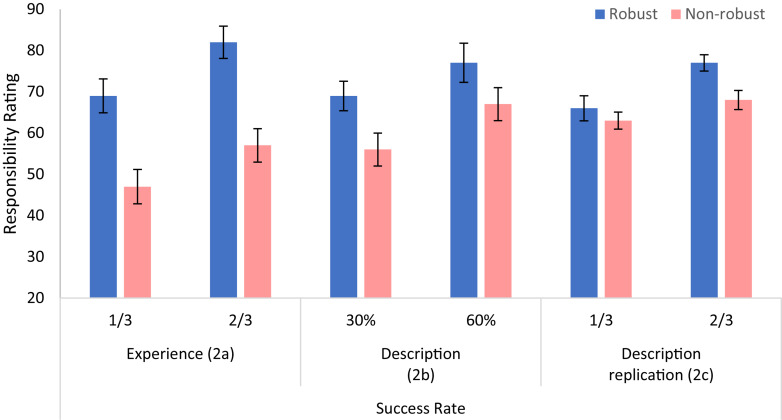
Judgments of causal responsibility, as a function of success rate and robustness, in Experiment 2, for the Experience (left panel, *n* = 22) and for the Description (middle panel, *n* = 20; and right panel, *n* = 46) conditions. Error bars indicate within-subject ±1 standard error of the mean.

The responsibility judgments are shown in Figure 4. Three 2 × 2 ANOVAs with robustness (robust vs. non-robust) and success-rate (1/3 vs. 2/3) as within-subject factors were carried out. In Experiment 2a, we find main effects of robustness and of success rate. The participants rated the players that took robust shots as more responsible for the goal than those that took non-robust shots, *F*(1,21) = 13.01, *p* = 0.002, $\eta_{\text{p}}^{2}$ = 0.408. Participants also rated players who had a high success rate more responsible for their goals, compared to players who had a low success rate, *F*(1,21) = 14.10, *p* = 0.001, $\eta_{\text{p}}^{2}$ = 0.426. There was no significant interaction between robustness and success rate, *F*(1,21) = 0.12, *p* = 0.733, $\eta_{\text{p}}^{2}$ = 0.006.

In Experiment 2b (description), participants also rated the robust players as more responsible for the goal than those that took non-robust shots, yet this effect was only marginally significant, *F*(1,19) = 3.06, *p* = 0.095, $\eta_{\text{p}}^{2}$ = 0.127. As in Experiment 2a there was a main effect for success rate, *F*(1,19) = 6.38, *p* = 0.020, $\eta_{\text{p}}^{2}$ = 0.233, and no interaction between robustness and success rate, *F*(1,19) = 0.26, *p* = 0.617, $\eta_{\text{p}}^{2}$ = 0.012. Because robustness was only marginally significant in the description condition, we performed a replication study with a larger number of participants (Experiment 2c; see Footnote 10). We replicated our findings. Participants rated the robust players as more responsible for the goal than those that took non-robust shots, *F*(1,45) = 4.24, *p* = 0.045, $\eta_{\text{p}}^{2}$ = 0.086. Participants also rated the more successful players as more responsible, *F*(1,45) = 8.19, *p* = 0.006, $\eta_{\text{p}}^{2}$ = 0.154, with no interaction between these two factors, *F*(1,45) = 1.42, *p* = 0.240, $\eta_{\text{p}}^{2}$ = 0.030.

### Discussion

The results indicate that both success rate and robustness (as operationalized by the action-outcome contingency being dependent on background conditions), independently affect causal responsibility ratings. Note that while the effect of robustness appears larger in the experience condition^11^, it is also present in the description condition. For the experience-based condition, one may argue that the participants make inferences on the success probabilities that are subject to bias in the priors, resulting in larger success rates for the curved (thus impressive) compared with the straight strategy kicks. Such biases could generate a robustness effect due to a success-rate artifact. Such a memory bias effect, however, is less plausible in the description condition, in which the success rates are explicitly stated (see Experiment 4 for an explicit test showing that the participants showed accurate memory of stated success rates).

The results indicate that the robustness of an action (the way the player takes the free-kick) affects the attribution of responsibility for its outcome (the goal), independent of the success rate. The results also consistent with the probability-raising principle, and indeed robust actions are typically more likely to succeed (but see Experiments 3–4 and Vasilyeva et al., 2018, for situations in which this is not the case). Both of these results could be understood to result from the negative influence of luck on the degree of responsibility, and from the idea that goals by players who shoot through the wall are more likely to have resulted from luck (cf. Gerstenberg et al., 2018).

There is, however, an alternative interpretation for the robustness effect. Accordingly, one may argue that the robust and successful kicks involve more skill, and therefore, it is a skill and not robustness *per se* that affected participants’ judgments. While the skill hypothesis was not supported in Experiment 1, Experiment 3 will control both probability-raising and skill.

## Experiment 3

In this experiment, we further test for robustness using a design that controls both probability-raising and skill. Our experimental set-up is inspired by the Ellsberg paradox (Ellsberg, 1961*;* see also Vasilyeva et al., 2018). Consider an agent that is faced with a lottery in which a ball is randomly selected from one of two urns (Table 2). In Urn 1 there are 30 red, 10 black, and 50 yellow balls while in Urn 2 there are 30 red, 50 black, and 10 yellow balls. The agent has no control over which urn is chosen; the urn is selected by another person. The agent also does not know the probability with which this person chooses Urn 1 versus Urn 2 and can bet either on red or on black. Which color should she bet on? The typical Ellsberg paradox result, is that when faced with such choices, agents prefer to bet on red (which has a definite 1/3 probability of a win) than on black (whose win probability is not determined and can be anything between 1/9 and 5/9). Here, we don’t focus on preferences, but rather on how observers judge the causal responsibility of agents who achieve a goal by taking a robust or a non-robust action.

**TABLE 2:** An adaptation of the Ellsberg paradox.

	Red balls	Black balls	Yellow balls
Urn 1	30	10	50
Urn 2	30	50	10

*The values indicate the number of differently colored balls in two different urns.*

Note, that for this situation, the outcome of the action (bet-red or bet-black) depends probabilistically on both the ball selected and on the background condition (the other person selecting Urn 1 or Urn 2). The bet-red action is thus more robust than bet-black, because its win probability is stable across background conditions (Urn 1 vs. Urn 2). By betting on red, one’s probability of success is rendered independent of the urn selection, whereas betting on black entails that one’s probability of success depends on the urn chosen by the other person.

According to the robustness hypothesis, we predicted that participants would judge that an agent who wins as a result of a robust bet is more responsible than one who wins as a result of an unstable bet. Note that here there is no difference in the skill that the execution of the strategy requires. In addition, we also manipulated whether the background condition (Urn 1 vs. Urn 2) was decided by another person or by a computer. We hypothesized that if the background circumstance involves another agent who acts intentionally, the robustness effect will be enhanced, compared with a background circumstance that involves a non-intentional mechanism (Lombrozo, 2010). One possibility, for example, is that the presence of an agent (as a background condition) makes this background condition more salient.

To make the setting more realistic for our student participants, we framed the task in an exam setting. In particular, participants were asked to rate a candidate’s responsibility for exam successes, with relation to his/her preparation for this exam. Participants read about six candidates, each of whom prepared differently in the way they allocated study time to the potential exam-topics (studied both topics or only one of them) and with regards to the total time they studied (duration of 3 or 5 days). The exam topic was picked randomly by the computer or by another agent. In this experiment, we were interested in responsibility for success in the exam. However, we added two additional filler candidates who failed the exam, in order to make the test more ecologically valid (it is unlikely that all candidates would be successful).

### Method

#### Participants

Twenty Tel Aviv university students (17 females, 3 males; mean age = 22.9, *SD* = 2.0) took part in return for 15 min credit points needed in their BA requirements. Subjects spoke Hebrew as their mother tongue (17), or had at least advanced Hebrew reading capabilities (3).

#### Materials and Procedure

The experiment was run in lab, and presented via Qualtrics. First, participants read the following short text that introduced a hypothetical hiring procedure (exam topics and success rates were bolded and colored to ease the tracking of details; see Supplementary C):

“Assume that Google is recruiting new employees who need to pass a knowledge and ability exam. The exam questions this year may involve one of two possible topics: Algorithms or Cryptography (Google informs the candidates about the possible topics 1 week before the exam). Assume also that a candidate who is good at programming and did a BA in Computer Science has a 30% chance to pass the exam without any special preparation, no matter what topic is tested. However, if the candidate studies for the exam, her/his chance to pass it will increase as follows:

• A candidate who studies for 3*days* on both topics (sharing time between them), will pass the exam with 50% chance if s/he gets a question about Algorithms, and also a 50% chance to pass if asked about Cryptography.
• A candidate who studies for *3 days* but chooses to learn only one topic, will pass with 70% chance if tested on this topic, but remains at 30% if asked about the other topic.
• A candidate who studies for *5 days* on both topics, will pass the exam with 60% chance if s/he gets a question about Algorithms, and also a 60% chance to pass if asked about Cryptography.
• A candidate who studies for *5 days* but chooses to learn only for one topic, will pass with 90% chance if tested on this topic, but remain at a 30% chance if asked about the other topic.

Next, participants were presented with information about a number of candidates for the latest Google-exam (see Table 3), all having “very similar intellectual abilities and programming skills, as reflected by their BA record, but differing in the way they prepared for the exam, and on the circumstances that determined the exam topic.” The participants were asked to evaluate the responsibility of each candidate for passing or failing the exam on a 1–100 slider scale bar (see Supplementary C). The candidates (and their evaluations) were presented sequentially (not in table format), and participants were told that they can take as long as they need and that they are allowed to look back and compare previous judgments.

**TABLE 3:** The candidates presented for judgment in Experiment 3.

Candidate	Robust
**A** studied for 3 days on both *Algorithms* and *Cryptography* and passes the exam.	+
**B** studied for 5 days on both *Algorithms* and *Cryptography* and passes the exam.	+
**C** studied for 3 days only on *Cryptography* and passes the exam.	−
**D** studied for 5 days only on *Cryptography* and passes the exam	−
**E** studied for 3 days only on *Algorithms* and passes the exam.	−
**F** studied for 5 days only on *Algorithms* and passes the exam.	−
**G** studied for 3 days on both *Algorithms* and *Cryptography* and fails the exam.	+
**H** studied for 5 days only on *Cryptography* and fails the exam.	−

Participants were told that “in the morning of the exam, the exam-topic of *Cryptography* was randomly chosen by the computer software.” After answering all of the eight evaluations, the participants were asked to make the same (A–H) evaluations again with one difference, which involved the manner in which the exam-topic was selected. Instead of having the topic randomly selected by the computer-software, participants were told that “in the morning of the exam, the topic of *Algorithms* was randomly chosen by computer software, but the head of recruitment decided not to let the computer determine the exam-topic and switched it to *Cryptography*.”

The reason for the second set of evaluations was twofold. First, we wanted to test if the effects (of success rate and of robustness) are stable. Second, we wanted to test if the robustness effect is modulated by the presence of another agent, who is involved in the setting of the background conditions that, together with the candidate’s action, determine the action’s success (a type of responsibility dilution). For each exam candidate, the participants were asked to rate “to what extent is the candidate responsible for his success/failure in the exam?” (A screenshot of the materials is presented in the Supplementary C).

#### Analysis

We focus on a number of contrasts, based on the candidates who passed the exam. We focus on the candidates that succeed in their exam, because our theory of robust causation depends on the agent taking an action that is intended to bring about an event (Woodward, 2006; Usher, 2018). Thus, robustness manipulations should be tested on events that match the agent’s intention (success cases) and not on events that do not match (failures); but see further discussion for the case of failure in Experiment 4.

First contrasting robust candidates (A and B) with non-robust candidates (C and D; see Table 3) provides an estimate of the robustness effect. Second, contrasting candidates A and C, who studied for 3 days, with candidates, B and D, who studied for 5 days, provides an estimate for the effect study-duration (3 vs. 5 days). Third, comparing candidates C and D, who passed by studying only the selected topic, with candidates E and F, who passed by studying the topic that was not selected, provides an estimate of the effect that the match between the topic studied and the one selected makes for non-robust type actions (this match affects the success rate, conditioned on the background condition that was active). For example, if one studies the topic that was probed, the success rate should be inferred to be higher, and we predict that this will affect the responsibility ratings for success in the two cases. Fourth, the difference between the robustness effect in the computer-condition and in the “head of recruitment” condition reveals whether the robustness effect is modulated by the presence of an agent.

### Results and Discussion

**FIGURE 5:**
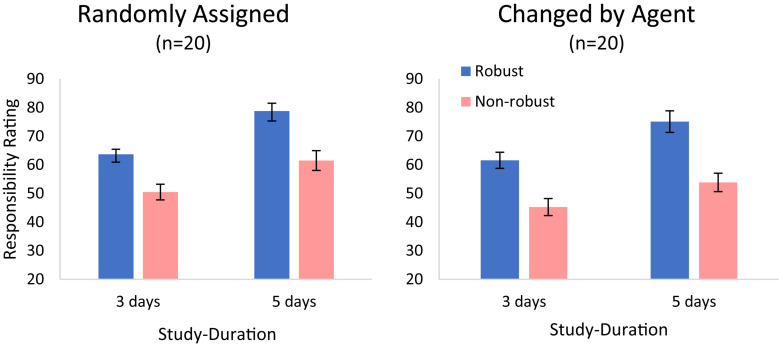
Ratings of responsibility (cases A–D, in Table 3) in Experiment 3. Both the study-duration and the robust/stable action received higher ratings. Error bars indicate within-subject ±1 standard error of the mean.

Planned comparisons provided significant differences for all the variables above. Specifically, there was a significant robustness effect, where responsibility ratings of robust candidates A and B (*M* = 69.82, *SD* = 17.21) were higher than of non-robust candidates C and D [*M* = 52.80, *SD* = 19.00; *F*(1,19) = 12.69, *p* = 0.002, $\eta_{\text{p}}^{2}$ = 0.400], and a significant study-duration effect, where responsibility ratings of the three-learning-days candidates A and C (*M* = 55.28, *SD* = 15.70) were lower than of five-learning-days candidates B and D [*M* = 67.34, *SD* = 14.27; *F*(1,19) = 69.29, *p* < 0.001, $\eta_{\text{p}}^{2}$ = 0.785; see Figure 5]. This is consistent with the 2 × 2 × 2 within subjects ANOVA (on the A–D items), which resulted in three main effects (robustness, success-rate, and agent-framing) but no significant interactions. The “head of recruitment” framing reduced responsibility judgments, *F*(1,19) = 5.61, *p* = 0.027, $\eta_{\text{p}}^{2}$ = 0.228. However, while this effect was numerically larger for the non-robust (C and D) than for the robust (A and B) candidates, this difference did not reach statistical significance, *F*(1,19) = 0.79, *p* = 0.384, $\eta_{\text{p}}^{2}$ = 0.040.

Finally, we examine the effect that the difference in match (between the topic studied and the one selected) makes for non-robust type actions (C and D vs. E and F). Planned comparisons revealed that participants rated the match cases higher (studied *Cryptography* and exam-topic was *Cryptography*) than non-match cases [studied *Algorithms* and exam-topic was *Cryptography F*(1,19) = 11.37, *p* = 0.003, $\eta_{\text{p}}^{2}$ = 0.374].

The results of this experiment confirmed most of our predictions. First, as predicted by both robustness and probability raising, participants gave higher responsibility ratings for exam success to candidates who had a higher chance of success as a result of studying more. However, as predicted by robustness alone, participants rated robust candidates higher, who studied both topics, and thus their success was less dependent on background conditions.

Nevertheless, it is possible to query the type of judgments that the participants made. While we formulated this in terms of the “to what extent is the agent responsible for success/failure in the exam,” one may also wonder whether participants distinguish between this and mere causal strength evaluation. Indeed, many experiments (including our Experiment 1) obtain similar results with causal strength and causal responsibility judgments. Our final experiment aims to contrast between these two measures and also to extend the judgments from cases of success to both success and failure.

## Experiment 4

In Experiment 4, we aimed to replicate the results of Experiment 3 (validating memory of the success rates) and to contrast judgments of responsibility and judgments of causal strength. Although often, these two types of judgments have parallel effects, we expect these judgments to come apart in this specific setup. Compare, for example, candidates A and C (see Table 3), both of whom succeeded in the exam after having studied the same amount, but with A having divided the study among the two topics, while C having studied, only the topic that was tested. Following Chockler and Halpern (2004), we proposed that when judging the extent to which each candidate is responsible for the exam’s success/failure, participants will take the *epistemic perspective* of the candidates at the time they made the action. On the other hand, when asked to evaluate the causal strength by which the action caused the effect, we expect participants to take an objective perspective, which includes the actual background circumstances. Indeed, in the actual situation in which the exam topic was chosen for which the non-robust candidate studied, the non-robust candidate has a greater success contingency than the robust-candidate.

In addition, we wanted to extend the range of cases to include cases of failure. For the case of responsibility, we do not make a specific prediction on how robustness (as expressed by studying a single or two topics) will affect the responsibility of failure (This is because failures do not satisfy the intentional-match requirement in robust action, and the robust action is more stable in its prediction of both success/failure). However, we expect a dissociation between the effects of study-duration on causal strength and responsibility judgments. Consider the case of an agent studying for only one topic, which does not come up in the exam, resulting in exam failure. While judgments of causal strength should be invariant to how long the agent studied (as the amount of studying the wrong topic should not affect the contingency with exam success/failure), judgments of causal responsibility are expected to decrease with study duration. Indeed, if judging responsibility depends on adopting the epistemic perspective of the agent, the actual background circumstances (topic mismatch) should not be assumed, and therefore, the more an agent studies for the exam, the more responsible she is for success (and less for failures), independent on whether the topic matches or not.

The experiment was identical to Experiment 3, except for a few modifications. First, we removed the head of recruitment vs. computer condition (we kept the computer framing only), and we included four candidates (A–D) that succeeded in the exam, and four candidates who failed (two who studied both topics, and two who studied the wrong topic). Second, we manipulated the type of rating (causal responsibility vs. causal strength) between participants in order to prevent a carryover between the two types of judgments. This allowed us to test the predicted dissociation between causal strength and responsibility judgments in cases of success, based on adopting the agent’s epistemic perspective in the latter. Finally, we also included a post-test memory check, in which we asked participants about the success rates of the various candidates, in order to ensure that participants based their judgments on the data we provided.

### Method

#### Participants

Sixty students at Tel Aviv University (30 in each condition^12^; 23 females, 37 males; mean age = 22.5, *SD* = 1.6) participated in this study in return for 15 min credit points.

#### Materials

The framing of the story was identical to Experiment 3. The eight candidates presented for evaluation are shown in Table 4.

**TABLE 4:** The job-candidates presented for judgment in Experiment 4.

Candidate	Robust
**A** studied for 3 days on both *Algorithms* and *Cryptography* and passes the exam.	+
**B** studied for 5 days on both *Algorithms* and *Cryptography* and passes the exam.	+
**C** studied for 3 days only on *Cryptography* and passes the exam.	−
**D** studied for 5 days only on *Cryptography* and passes the exam	−
**E** studied for 3 days on both *Algorithms* and *Cryptography* and fails the exam.	+
**F** studied for 5 days on both *Algorithms* and *Cryptography* and fails the exam.	+
**G** studied for 3 days only on *Algorithms* and fails the exam.	−
**H** studied for 5 days only on *Algorithms* and fails the exam.	−

#### Procedure

Responsibility judgments were assessed in the same way as in Experiment 3. In the causal strength condition, participants were asked: “To what extent did the study of the candidate cause the outcome in the exam?” As a memory check, after judging the candidates, participants were asked to fill in a table with success rates of the various candidates.

#### Analysis

Based on the predictions we outlined, we carried out 2 × 2 ANOVAs for passing candidates with factors of robustness and study-duration, separately for each judgment type (responsibility vs. causation). While cases of failure do not satisfy the intentional match criterion above, we also report the responsibility for these judgments, and we carry out a similar 2 × 2 × 2 ANOVA for the cases of failure.

### Results and Discussion

The post-experimental memory test showed that the participants remembered well the success rates of the eight candidates that they were required to rate, as indicated by the post-experimental memory test (see Supplementary Figure S2 and Supplementary C). We now turn to the ratings of causal responsibility and of causal strength.

#### Causal Responsibility

**FIGURE 6:**
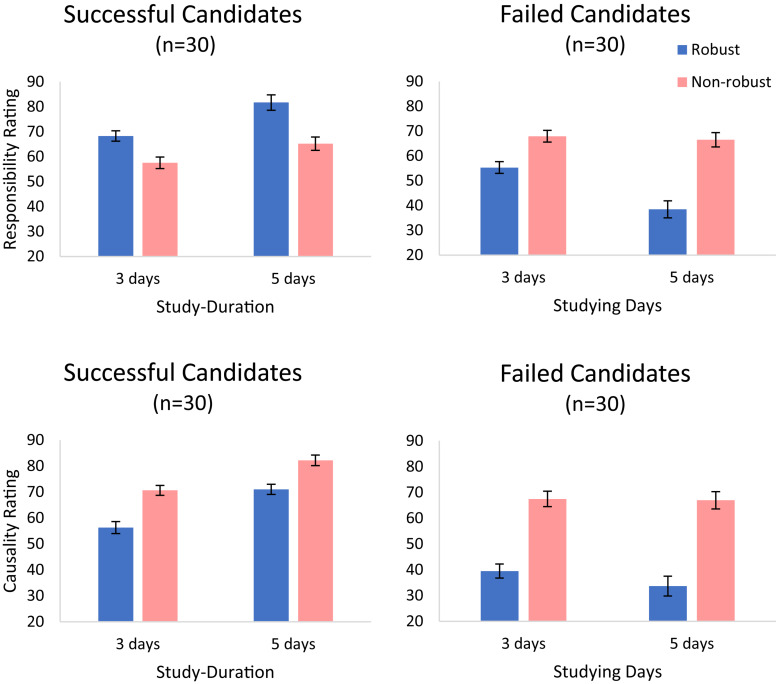
Responsibility ratings (upper panels) and causal strength ratings (lower panels) for passed (left) and failed (right) candidates in Experiment 4. Error bars indicate within-subject ±1 standard error of the mean.

For successful candidates (A–D in Table 4), we replicated the results of Experiment 3. There were two main effects, for robustness [*F*(1,29) = 8.50, *p* = 0.007, $\eta_{\text{p}}^{2}$ = 0.227] and for study-duration [*F*(1,29) = 34.62, *p* < 0.001, $\eta_{\text{p}}^{2}$ = 0.544], respectively. As shown in the upper-left panel of Figure 6, participants gave higher ratings to the robust study candidates and also to candidates who studied longer. There was also an interaction between these two factors [*F*(1,29) = 5.46, *p* = 0.027, $\eta_{\text{p}}^{2}$ = 0.158]. However, the simple effects of robustness were significant at both study-duration conditions [for 3 days, *F*(1,29) = 7.09, *p* = 0.012; for 5 days *F*(1,29) = 8.94, *p* = 0.006].

For the failed candidates (E–H in Table 4; upper-right panel in Figure 6), we obtained a main effect of robustness. Participants rated the candidates who studied in a robust way (both topics) as less responsible for their failure (*M* = 46.89, *SD* = 25.97) than the ones who studied in a non-robust way [on the topic that was not chosen, *M* = 67.23, *SD* = 23.52; *F*(1,29) = 18.70, *p* < 0.001, $\eta_{\text{p}}^{2}$ = 0.392]. We also found a main effect of study-duration [*F*(1,29) = 14.36, *p* = 0.001, $\eta_{\text{p}}^{2}$ = 0.331] and an interaction between robustness and study-duration [*F*(1,29) = 17.00, *p* < 0.001, $\eta_{\text{p}}^{2}$ = 0.370]. The duration of study reduced the responsibility for failing in the exam *only* for the candidates that studied both topics [*F*(1,29) = 22.13, *p* < 0.001, $\eta_{\text{p}}^{2}$ = 0.433].

#### Causal Strength

For successful candidates, judgments of causal strength showed main effects of robustness and study-duration. As in the responsibility condition, the duration of study increased the ratings [*F*(1,29) = 153.72, *p* < 0.001, $\eta_{\text{p}}^{2}$ = 0.841; lower-right panel]. However, oppositely to the responsibility condition, here robustness strongly *decreased* causal strength ratings [*F*(1,29) = 11.55, *p* = 0.002, $\eta_{\text{p}}^{2}$ = 0.285]. Indeed, participants judged that a candidate who spent her time studying only the topic that was selected, caused the success in the exam to a higher degree (*M* = 76.36, *SD* = 21.05) than one who spent the same amount of time studying both topics (*M* = 63.61, *SD* = 19.94). Thus, judgments of causal-strength, but not of causal responsibility, appear to track the extent to which the action increased the probability of the outcome (conditional on the actual background conditions).^13^ There was no interaction between study-duration and robustness [*F*(1,29) = 1.62, *p* = 0.213, $\eta_{\text{p}}^{2}$ = 0.053].

Finally, for failed candidates there was a main effect of robustness. Participants saw non-robust candidates who studied the wrong topic to have caused their failure to a higher degree (*M* = 67.20, *SD* = 29.44) than robust candidates who studied both topics [*M* = 36.58, *SD* = 19.39; *F*(1,29) = 24.74, *p* < 0.001, $\eta_{\text{p}}^{2}$ = 0.460]. Also, like for casual responsibility, the amount of study reduced casual strength [*F*(1,29) = 4.75, *p* = 0.037, $\eta_{\text{p}}^{2}$ = 0.141]. This reduction seems to be stronger in candidates who studied both topics, but this interaction (between robustness and study-duration) was not significant [*F*(1,29) = 2.50, *p* = 0.125, $\eta_{\text{p}}^{2}$ = 0.079].

## General Discussion

In four experiments, we tested if the extent to which agents are held causally responsible for the outcomes of their actions is affected by the robustness with which the action brought about the outcome (Woodward, 2006; Icard et al., 2017; Vasilyeva et al., 2018). In the first experiment, we manipulated robustness by explicitly increasing the number of background circumstances (possible dice outcomes) in which the action (dart throw) results in a win. We did this by contrasting actions (dart throws) that result in one, two or three dice rolls, depending on the ring number of the dart on the board (where success requires a sum of 6 on the dice). We found that agents whose dart throws result in more dice are seen as more responsible for success (and less responsible for failures). We observed the same pattern of results for judgments of causal strength. The parallel effects of robustness on judgments of causal strength and causal responsibility are consistent with the responsibility-view, which sees causal attributions as mediated by normative attributions about what an agent should have done in a given situation (Sytsma et al., 2012). To achieve more dice rolls, one needs more skill. However, by comparing even vs. odd ring-numbers, we found that participants’ judgments of causal responsibility and causal strength tracked robustness and were not merely affected by the skill of the player. These results are also consistent with *probability-raising* accounts according to which responsibility and causal strength judgments track the extent to which an action increased the probability of the observed outcome (Suppes, 1970; Cheng and Novick, 1992; Spellman, 1997; see also Kominsky et al., 2015; Icard et al., 2017, for recent studies showing that judgments of causal strength vary with the typicality of the cause and the background conditions).

Experiments 2–4 tested for effects of robustness while controlling for probability raising. Experiment 2 examined a soccer scenario in which strikers had two different ways of taking free-kicks. The non-robust action is to shoot the ball directly through the defensive wall. Such an action may result in a goal, depending on background circumstances that the agent does not control (the position and movement of the defending players). The robust action is to bend the ball around the wall; if well-executed, this action results in a goal in a way that depends less on background conditions (the exact location of the defenders do not matter). Because taking a well-executed curved kick is difficult, players may have similar success rates when employing the two strategies. We thus presented participants with animations of such hypothetical players, and independently manipulated success probability and robustness. To ensure that participants are not biased in their success probability assessment by the type of kick (due to prior expectations), we included a condition that stated the success probability, rather than leaving it for participants to estimate. For both description and experience conditions, we found that ratings of responsibility increased with robustness, even when the probability of success was kept constant. While these results support the robustness hypothesis, they are subject to an alternative explanation. In particular, it is possible that the ratings don’t reflect considerations of robustness, but rather the inferred skill of the agent (cf. Gerstenberg et al., 2018). Experiments 3–4 addressed this issue using a scenario based on an Ellsberg-type design and using an exam-success setting (see also Vasilyeva et al., 2018, for a similar design).

In Experiment 3, the action (the way to prepare for the exam) did not vary in skill between the robust (split study time between both topics) and non-robust (study only one topic) action. Also, the overall success rate of the robust action was equated with that of the non-robust one, but the outcome of the non-robust action was more variable, depending on an external factor (selected exam topic). Hence, an account based on probability raising would not predict any differences in judgments. Note, moreover, that after a particular exam topic was selected the probability of success is now in favor of the non-robust case if the topic selected matches the one that the candidate prepared for. Like in Experiment 2, the results showed effects of both success rate and robustness. In particular, participants judged candidates who prepared for both topics more responsible for their exam success than those who only prepared for a single one, and were lucky in that this topic was chosen. Note also that judgments of responsibility tracked robustness even though, as stated above, the non-robust candidates actually had a higher probability of success given the lucky background.

Finally, in Experiment 4, we replicated the results of Experiment 3 under two important modifications. First, in addition to assessing causal responsibility, we also assessed judgments of causal strength. We predicted that the role of robustness would be different in judgments of causal responsibility versus causal strength, because the agent’s epistemic perspective is more important for judgments of causal responsibility (in this case, we predicted that participants would adopt the agent’s epistemic perspective). The results fully replicated the results of Experiment 3 in the responsibility/success case. Second, we examined how study-duration and study-type affect the responsibility and the causal strength judgments in cases of failures. Consistent with previous findings (Gerstenberg et al., 2018), participants judged agents who took robust actions that yielded more stable success rate, to be more responsible in case of success, but less responsible in cases of failure. Note that while this result is easy to motivate for study-duration (because it is positively correlated with success-rate and negatively correlated with failure-rate), it is less straightforward for study-type. Here the robust action, of studying both topics, is more stable with regards to both the success/failure events. In other words, while a robust action that resulted in success is more stable to changes in background conditions, so is a robust action that resulted in failure. These results are consistent with the idea that the effect of robustness (studying one vs. both topics in Experiments 3–4) is evaluated based on the stability of the contingency between the action and the successful outcome (the action’s goal). We propose that for cases of failure, the robustness is derived from a negation of the goal-achievement: because the agent is more responsible (when doing A compared with B) if she succeeded in achieving her goal, she is less responsible (when doing A compared with B), in case the goal was not achieved. Finally, we find that for cases of failure, neither causal responsibility nor causal strength are affected by the study duration, for candidates who study only the wrong topic. This shows that study duration alone affects causal judgments only when it plays a role in the causal chain of events from action to outcome.

While we attribute the robustness boost of the responsibility judgments in Experiments 2–4 to the lack of causal dependency on background conditions (external to the agent), such as other players (Experiment 2) or the exam selection (Experiments 3–4), it is still possible to suggest that some of the participants are nevertheless responsive to some inferred agential trait, such as skill (Experiment 2), or study efficiency (Experiments 3–4). We ruled out skill as a mediator, in our analysis of Experiment 1, and our instructions attempted to eliminate it as a factor in Experiments 2–4. For example, we explained that the success rate of robust/non-robust players is the same (Experiment 2), and that all candidates are equal in their computer science knowledge and abilities (Experiment 3–4). Nevertheless, it is still possible that some participants may have inferred that a robust candidate (who studied both topics and succeeded) is more effective than a non-robust one (who studied the one topic that happened to be assessed in the exam). Accordingly, as suggested by Gerstenberg et al. (2018) an inferred agent trait (skill) could potentially mediate the effect that robustness has on judgments of causal responsibility. While future studies will be needed to test the possibility of further dissociating robustness from skill, we believe that this should not be viewed as a confound. Rather skill is probably a necessary feature of agents who exercise robust control (Usher, 2018).

The most novel result of this experiment, however, was the difference between the patterns observed in the causal responsibility and the causal strength condition (see Figure 6, left panels). While the causal responsibility of the agent increased with robustness (as the participants took the agent epistemic perspective), the causal-strength decreased with robustness, as the participants took a more objective perspective, assuming knowledge of the actual background circumstance. We believe that this dissociation (and deviation from the responsibility view; Sytsma et al., 2012), which is a rare one in the literature, was made possible by the specific Ellsberg-type design, which allowed us to dissociate between the objective contingency and the agent-based epistemic one. While future studies will be needed to test the possibility of further dissociating robustness from inferred skill in this type of design, we believe that in ecological conditions (e.g., Experiment 2) skill and robustness are associated, as skill is typically a feature agents need to deploy in order to exercise robust control (Usher, 2018). In the following, we discuss the implications of our results for normativity and their relation with other related studies.

### Normativity

The results of Experiment 1 are readily understood from a normative perspective: rational agents should aspire to increase the likelihood of their desired outcomes. As discussed by Woodward (2006) robust actions (shooting a person in the heart) are more likely to achieve a goal (like the death of the victim), compared to non-robust actions (shooting the victim in the leg, which may or may not result in death), as the outcomes of such non-robust actions are likely to depend on background circumstances. Similarly, Woodward (2006) has argued that robustness is a critical difference that distinguishes between cases of causation by action and causation by omission or by double prevention (see Lombrozo, 2010 and Cushman and Young, 2011, for experimental studies showing that participants are sensitive to these differences in their causal judgments). Furthermore, Lombrozo (2010) has argued that moral judgments are affected by the stability of the causal relation to variations in background circumstances. More recently, Usher (2018) has argued that in order to achieve robust causation of actions over desired outcomes, agents deploy a teleological guidance control that is based on a means-ends strategies (cf. Heider, 1958). As agents do not have access to all information on background circumstances, they should attempt to act so as to make the outcome less dependent on such circumstances. In our Experiment 1, achieving a 3 dice roll, grants the agent with more opportunities to succeed, making her less dependent on chance. A similar situation obtains in Experiment 2, by attempting a curved-style free-kick, the agent takes an action whose outcome is less dependent on circumstances beyond her control.

In Experiments 2–4, we clarified to our participants that the probability of success of the robust and non-robust action is the same. In Experiment 2, robustness to background circumstances was balanced by the difficulty of executing such an action. In Experiments 3–4, were inspired by Ellsberg scenario (Ellsberg, 1961) that let us keep the probability of success fixed but to vary the robustness. Still, we find that people evaluate the agent as more responsible for the outcome of her action, in the case of robust action. The normativity of this judgment, thus, requires a special discussion.

Our conceptualization of robustness, via a count of background circumstances that enable an intended event was proposed by Woodward based on a number of conceptual considerations, such as invariance (Woodward, 2003, 2006). This conceptualization also has the advantage that it does not require access to probabilities of the background circumstances (which are often difficult to access). Usually, robustness as measured by this count definition, correlates with the success probability as in our Experiment 1, however, robustness and probability raising can also stand in opposition. For example, one may contrast an action that produces an effect in 10 background conditions with a small probability (say, 5% in each) with another that produces the effect in a single background condition (but with a higher probability, of say, 90%). It is beyond our aim to make either normative or empirical claims about what is expected in such special situations. Our Experiments 2–4, kept the total success probability fixed while varying the count-type robustness. Thus, we believe they support the more modest conclusion, that once the success probability is fixed, the count-measure of robustness affects the judgments of causal responsibility. Future investigations will be required to examine tradeoffs between success probability and count-measures of robustness.

What our Experiments 3–4 show is that the preference for the stable alternatives (those whose success rate does not depend on factors that are not known, also labeled as *ambiguity-aversion*; Ellsberg, 1961), is also reflected when we judge agents who take robust actions (in the sense above) as higher in causal responsibility. We believe that the reason for this is the fact that the success of the non-robust action appears lucky (see also Gerstenberg et al., 2018), as it depends more on other agents or circumstances. In Gerstenberg et al. (2018), the contrast between agent-bearing responsibility actions (for which the agent gets high credit) and lucky ones (for which she gets less credit) was made via the contingency between the action and the outcome. Here, we kept this contingency constant (or even reduced it in the case of robust actions compared to non-robust matched actions, Experiments 3–4), but we manipulated the presence of non-agent background conditions. Thus, consistent with Woodward’s theory of robust causation (2006), lucky actions are those in which the background conditions contributed significantly to the outcome, and thus, non-robust actions receive lower responsibility, reflecting a type of diffusion of responsibility among multiple causes (Lagnado et al., 2013). Indeed, in previous studies, Lagnado et al. (2013) showed that when the number of agents that disjunctively contributed to an event increases, the judged responsibility of each agent is reduced. Since our non-robust actions allow other agents (or factors) to contribute to the production of the intended event (note that this may involve a contribution by non-acting, as for the defenders who miss blocking the ball shot through the wall), we can think of their effect on judgments of responsibility as a type of responsibility diffusion. The robust actions, on the other hand, are such that they screen-off the intended event from the impact of other agents or background circumstances and thus, they satisfy a robust sufficiency criterion.

Finally, another advantage of robustness is that robust causal setups have the advantage that the causal Markov condition^14^ is preserved on the level of overall categories. In contrast, in setups with subcategories with the same causal structure but different causal strength, the Markov condition does not hold globally, resulting in distorted judgments of correlations and causal relationships (von Sydow et al., 2016; Hebbelmann and von Sydow, 2017; cf. Hagmayer et al., 2011).

### Relation to Other Work and Alternative Theories

In a recent paper, Vasilyeva et al. (2018) reported that people’s judgments about causal generalizations and causal explanations are sensitive to the stability of these relations, even when probability-raising is controlled. In their studies, participants were presented with descriptions or contingency tables for a potential causal relation, and were then asked to indicate the degree to which they endorse a causal explanation (or causal generalization) for the situation described. For example, in studies 1 and 2, participants were presented with contingency tables for fictional lizard-like species (Zelmos), which either did or did not eat yona-plants (the action) and either did or did not get sore antennas (the effect). These tables included a moderating variable (drinking salty/fresh water) that varied or did not vary the relationship between the action and the effect. The presence of the moderating variable that affected the action-effect relationship reduced the degree of causal endorsement, even though the average causal strength across the moderating variable was the same. In their study 3, a similar result was obtained for the endorsement of a causal relation between people taking a vitamin and the effects on bone density, with gene-type as a moderating variable.

While these findings parallel our results from Experiment 3, there are a number of important differences, and thus we believe that the two approaches complement each other. The central difference is that while our experiment was designed to assess people’s judgments of the extent to which an agent’s action was causally responsible for bringing about an outcome they intended, Vasilyeva et al. (2018) assessed people’s endorsement of causal relations and of causal explanations between (type or token) events.

For example, in one of their experimental conditions, which is most similar to ours, after being presented with the background and the contingency tables, participants were told: “Your assistants select one of the zelmos with sore antennas from your second experiment. They call him Timmy. During the experiment, Timmy has eaten yonas. *You do not know whether Timmy drank fresh water or salty water during the experiment*. How much do you agree with the following statement about what caused Timmy’s sore antennas? *Eating yonas caused Timmy’s antennas to become sore*” (Vasilyeva et al., 2018, Table 2, p. 8).

Compare this with our scenario in Experiments 3 and 4, where participants evaluated the causal responsibility that the agent’s action (type of study) has for the outcome (success/failure in exam), under conditions that differ in sensitivity to an external circumstance (question chosen by the computer or by another agent). There are two important differences. First, as we formulate this at the level of agents taking an action toward a goal, we can probe the causal responsibility of the agent for the outcome of the action and contrast it with the causal-strength (it would make little sense to ask “how responsible is the Zelmo for getting sore antennas” in this context); agent-responsibility requires a set of minimum epistemic conditions, such as the agent foreseeing the potential consequences of her actions (or being in a state where she is expected to do so), which are in place in our case. While one may ask instead about the causal responsibility between the events (‘eating yonas’ and ‘having sore antennas’), we point below to an important difference.

Second, there is an important epistemic difference. In the ‘zelmos sore-antenna’ case the reduced causal endorsement of the causal relation in the non-stable condition is conditioned on lack of knowledge: participants did not know about the state of the moderating variable (“*You do not know whether Timmy drank fresh water or salty water during the experiment*”). In our Experiment 3–4, on the other hand, participants knew the state of the background variable (the exam topic selected). In contrast to Vasilyeva et al. (2018), we obtained an increased degree of causal strength (in the non-robust condition), showing that when such information becomes available, the participants rely on it in their causal strength judgments, and they do not adopt the agent’s epistemic perspective [see Endnote 4, in Vasilyeva et al. (2018), for a similar result]. Both of these results are normatively reasonable, as it makes sense to attribute increased causal strength to a causal relation that has a stronger contingency (our Experiment 4, and results reported in Vasilyeva et al., 2018, Endnote 4), and also to feel uncertain of the causal relation (Vasilyeva et al., 2018) given lack of knowledge on whether the sample belongs to a case that does or does not involve causal relation.

More importantly, we find that, even in a condition in which the causal contingency favors the non-robust action, causal responsibility judgments show a robustness effect: higher ratings for the robust actions. This provides a strong demonstration that robust actions confer more responsibility on an agent, even if the actual state of the environment happens to be such that the actual probability of success is lower. We have argued that the difference between opposed effects of robustness on causal responsibility and causal strength (Figure 6), stem from the fact than in responsibility attributions, participants consider the epistemic state of the agent. We believe that, taken together, our studies and those of Vasilyeva et al. (2018), provide compelling and complementary evidence for the importance of robustness in the endorsement of causal responsibility relations between events and of causal explanation, and in judging the causal responsibility an agent has for the outcome of her action.

### Further Implications and Future Research

We have focused here on judgments of *causal* responsibility. Future research is needed to clarify the normative aspect of stability in responsibility judgments, as well as its derivation from theoretical principles (e.g., Halpern and Hitchcock, 2015). Moreover, it has been argued that causal responsibility is a central component of legal and moral responsibility (Tadros, 2005; Moore, 2009; Lagnado and Gerstenberg, 2017; Usher, 2018). For example, it has been proposed that the degree of responsibility an agent has toward outcomes of her action depends on the teleological control that she deploys to achieve that effect (Lombrozo, 2010; Usher, 2018) and that differences in robust causation are the source of our feelings of a reduced responsibility toward manipulated agents (Deery and Nahmias, 2017; Murray and Lombrozo, 2017; Usher, 2018). Future research needs to test potential dissociations between robustness and skill and also examine how the attributions of responsibility change for teleological continual actions, in which the agent acts so as to carry out compensatory corrections needed to preserve a goal in the face of perturbations or interventions (Heider, 1958; Usher, 2018). Future research is also needed to examine potential distinctions between the causal responsibility of agents for the outcome of their intended actions (of the types we have examined here) and judgments of praise or blame.

In sum, robustness is an important but relatively under-explored causal concept (Woodward, 2006). Convergent evidence from our current studies, and also from Vasilyeva et al. (2018) using different experimental paradigms, show that robustness is itself a robust phenomenon in shaping people’s causal judgments.

## Data Availability Statement

All datasets generated for this study are included in the article/Supplementary Material.

## Ethics Statement

The studies involving human participants were reviewed and approved by Experiment 1: Ethics Committee, UCL Experiments 2–4: Ethics Committee Tel Aviv University (1321253). The participants provided their written informed consent to participate in this study.

## Author Contributions

MU and DL conceived the idea. DL, MU, and TG designed Experiment 1. GG and MU designed Experiments 2–4. GG ran Experiments 2–4. GG ran all analyses. MU, DL, and GG wrote the manuscript. All authors read the manuscript and contributed to improving it.

## Conflict of Interest

The authors declare that the research was conducted in the absence of any commercial or financial relationships that could be construed as a potential conflict of interest.

## Footnotes

1 These two causal responsibility patterns are as follows: (i) Harm brought about by an action is deemed morally worse than harm brought about by an omission. (ii) People judge harm used as the necessary means to a goal to be worse than harm produced as the foreseen side-effect of a goal (Cushman and Young, 2011).

2 The executive’s widow sued for compensation, but it was ruled that the negligence of lighting the match was not a cause of his death.

3 As the baseline recovery probability (without treatment) is fixed, the two treatments also have the same impact in terms of ΔP and causal-power (Cheng, 1997).

4 As discussed in Woodward (2003), there are a variety of invariance conditions, which a causal claim might satisfy. Robustness in the sense of invariance under changes in background conditions is just one such requirement. Other invariance requirements may be found, for example, in the characterization of causal power (Cheng, 1997), which rests on the assumption that causes will (at least in simple cases) conform to two invariance requirements — the power of the cause to produce an effect should be invariant under changes in the frequency with which the cause occurs and also invariant under changes in the frequency with which other causes of the effect occur.

5 Variations in this component can explain important differences between blame and causal responsibility. For example, unlike an adult, a child who pokes at a gun’s trigger out of curiosity will not be held culpable for resulting injury or death, given reasonable assumptions about the lack of relevant intentions (Shafer, 2000; Lagnado and Channon, 2008).

6 There are many contexts in which it is unclear how to count the “number of background conditions” (see Phillips and Cushman, 2017). In such contexts, the notion of robustness is not uniquely defined. However, in Experiment 1, there is a very natural way of counting the number of relevant background conditions. The number of background conditions is also well-defined when one set of background circumstances is a proper subset of another.

7 According to Woodward’s conceptualization, robustness depends on the number of background conditions (BC) under which the desired outcome takes place, given the action (Woodward, 2006). In this case, the relevant BCs are the possible outcomes of the dice that equal or exceed 6.

8 One may contend that a perfectly skilled and rational agent should be indifferent to differences between rings that are equivalent in the number of dice deployed. Such an agent, however, should always hit rings 5–6, and thus is inconsistent with most cases shown in the experiment. A more plausible interpretation of skill is to accept some unavoidable noise in the outcome and thus aim as close to the center as possible.

9 Experience based manipulations are more similar to real life, but they could be subject to biases in priors. The description-based manipulation explicitly states the success probability rather than relying on participants’ inferences from observation. Experiment 2c is a replication of Experiment 2b, which was carried out during the review of the manuscript.

10 We replicated Experiment 2b with a sample-size of 46 which was based on a power analysis that resulted in 80% power for the robustness effect of Experiment 2b.

11 It is possible that the larger effects in Experiment 2a are due to most participants being tested under more controlled lab conditions rather than online.

12 Based on the effect size in Experiment 3, this sample should allow a 95% power for replication of the robustness effect.

13 See Endnote 4 in Vasilyeva et al. (2018), for results showing that judgments of causal strength track the extent to which the action increased the probability of the outcome (conditional on the actual background condition).

14 The causal Markov condition is a critical assumption of the Causal Bayesian framework (Pearl, 2000; Woodward, 2003)– whereby any variable in a causal model is conditionally independent of its non-descendants given its direct causes. While there is controversy about whether people uphold this condition in their intuitive inferences (see Sloman and Lagnado, 2015, for summary), it is a desirable property for normative inference.
