# Supplementary material for: Causal Responsibility and Robust Causation
Source: Front Psychol. 2020 May 27;11:1069. doi: 10.3389/fpsyg.2020.01069 (PMC7269104; doi:10.3389/fpsyg.2020.01069)
Supplement: Supplementary file 1 [file Data_Sheet_1.docx]

**Causal Responsibility and Robust Causation**

Guy Grinfeld,^1^ David Lagnado,^2^ Tobias Gerstenberg,^3^ James F. Woodward^4^ & Marius Usher^1,5^

**Supplementary Materials**

All analysis codes and data files are uploaded on:

<https://github.com/guygrinfeld/Responsibility-and-Robust-Causation-Experiments>

1. **Additional results for Experiment-1**

A.*1* *Non-significant results in success-cases ANOVA.*

The ANOVA with number of dice rolled (one/two/three) and the ring-parity (odd vs. even) as within-subject factors, and judgment type (causation vs. responsibility) as a between-subject factor, results in non-significant effects for the ring-parity, *F*(1, 100) = 0.16, *p* = .690, η_p_ ^2^ = .002; judgment type, *F*(1, 100) = 0.01, *p* = .914, η_p_ ^2^ < .001) and no significant interactions (number of dice rolled and type of judgment, *F*(2, 200) = 0.18, *p* = .840, η_p_ ^2^ = .002; number of dice rolled and ring-parity, *F*(2, 200) = 0.22, *p* = .802, η_p_ ^2^ = .002; ring-parity and type of judgment, *F*(1, 100) = 0.62, *p* = .432, η_p_ ^2^ = .006; number of dice rolled, ring-parity and type of judgment, *F*(2, 200) = 0.28, *p* = .754, η_p_ ^2^ = .003 ).

*A.2 Regression models for success cases in Experiment-1*

The regression reported in Exp-1, is based on two mixed regression models with subjects as a random factor. The first model was done for the responsibility ratings and the second for causation ratings.

*A.2.1 Regression model for responsibility rating of success:*

Responsibility rating=b1*dice + b2*score + b3*ring + b_i_ *subject

|  | Estimate | Std.Error | t-value | | | | | | p-value |
| --- | --- | --- | --- | --- | --- | --- | --- | --- | --- |
| b_i_ | 22.744 | 11.685 | 1.946 | | | | | | 0.052 |
| b_1_ | 13.704 | 3.145 | 4.357 | | | | | | >0.001 |
| b_2_ | 0.492 | 0.347 | 1.418 | | | | | | 0.157 |
| b_3_ | 1.204 | 1.471 | 0.818 | | | | | | 0.414 |
|  | | | |  |  |  |  |  |  |

This model produces the following predictions:

Table S1

*Predicting responsibility rating*

|  | ring | dice | score | mean rating | mean prediction |
| --- | --- | --- | --- | --- | --- |
| 1 | 6 | 3 | 6 | 68.1 | 68.01 |
| 2 | 6 | 3 | 12 | 70.15 | 70.96 |
| 3 | 5 | 3 | 6 | 69.21 | 69.21 |
| 4 | 5 | 3 | 12 | 70.1 | 72.16 |
| 5 | 4 | 2 | 6 | 56.81 | 56.71 |
| 6 | 4 | 2 | 9 | 60.15 | 58.19 |
| 7 | 3 | 2 | 6 | 57.65 | 57.92 |
| 8 | 3 | 2 | 9 | 63.17 | 59.39 |
| 9 | 2 | 1 | 6 | 44.08 | 45.42 |
| 10 | 1 | 1 | 6 | 45.17 | 46.62 |

*A.2.1 Regression model for causation rating of success:*

causation rating=b1*dice + b2*score + b3*ring + b_i_ *subject

|  | Estimate | Std.Error | t-value | p-value |
| --- | --- | --- | --- | --- |
| b_i_ | 29.387 | 11.944 | 2.460 | 0.014 |
| b_1_ | 13.803 | 3.219 | 4.287 | >0.001 |
| b_2_ | -0.213 | 0.355 | -0.601 | 0.548 |
| b_3_ | 0.472 | 1.506 | 0.313 | 0.754 |

This model produces the following predictions:

Table C2

*Predicting causation rating*

|  | ring | dice | score | mean rating | mean prediction |
| --- | --- | --- | --- | --- | --- |
| 1 | 6 | 3 | 6 | 70.6 | 69.99 |
| 2 | 6 | 3 | 12 | 68.5 | 68.71 |
| 3 | 5 | 3 | 6 | 70.7 | 70.46 |
| 4 | 5 | 3 | 12 | 68.76 | 69.18 |
| 5 | 4 | 2 | 6 | 55.98 | 57.13 |
| 6 | 4 | 2 | 9 | 55.8 | 56.49 |
| 7 | 3 | 2 | 6 | 57.04 | 57.6 |
| 8 | 3 | 2 | 9 | 58.9 | 56.96 |
| 9 | 2 | 1 | 6 | 45.7 | 44.27 |
| 10 | 1 | 1 | 6 | 43.54 | 44.74 |

*A.3 Regression models for failure cases in Experiment-1*

We carried out additional analyses for the cases of failure (in which the score was below 6).

First, we present two mixed regression models with subjects as a random factor, equivalents to the ones above. Below we present the results for the critical case of score 5, which was resulted evenly in all dice-rolled outcomes.

*A.3.1 Regression model for responsibility rating of failure:*

Responsibility rating=b1*dice + b2*score + b3*ring + b_i_ *subject

|  | Estimate | Std.Error | t-value | p-value |
| --- | --- | --- | --- | --- |
| b_i_ | 69.63 | 11.643 | 5.981 | >0.001 |
| b_1_ | -11.021 | 3.116 | -3.537 | >0.001 |
| b_2_ | 0.307 | 0.529 | 0.580 | 0.562 |
| b_3_ | -0.281 | 1.469 | -0.8191 | 0.848 |

*A.3.2 Regression model for causation rating of failure:*

causation rating=b1*dice + b2*score + b3*ring + b_i_ *subject

|  | Estimate | Std.Error | t-value | p-value |
| --- | --- | --- | --- | --- |
| b_i_ | 74.965 | 12.518 | 5.989 | >0.001 |
| b_1_ | -8.258 | 3.371 | -2.450 | 0.015 |
| b_2_ | -0.261 | 0.572 | -0.456 | 0.649 |
| b_3_ | -1.816 | 1.589 | -1.143 | 0.254 |

*A 3.3 ANOVA for cases of failure*

We carried out ANOVA for the causation ratings of failure cases of score 5 (Figure S1, left panel) with number of dice rolled (one/two/three) and the ring-parity (odd vs. even) as within-subject factors. We found significant effects for the number of dice rolled , *F*(1, 98) = 3.12, *p* = .046, η_p_ ^2^ = .060, and for ring parity, *F*(1, 49) = 5.48, *p* = .024, η_p_ ^2^ = .100. The interaction between number of dice rolled and parity was not significant, *F*(2, 98) = 0.78, *p* = .460, η_p_ ^2^ = .016.

The ANOVA for the responsibility ratings of failure cases of score 5 (Figure S1, right panel) with same factors as above found significant effect only for the number of dice rolled , *F*(1, 102) = 17.24, *p* < .001, η_p_ ^2^ = .253. Ring parity, *F*(1, 51) = 0.36, *p* = .550, η_p_ ^2^ = .007, and the interaction between number of dice rolled and parity, *F*(2, 102) = 2.31, *p* = .104, η_p_ ^2^ = .043, were not significant.

*Figure S1.* Evaluations of causal strength (left) and of responsibility (right panel) for failure case of score 5, as a function of the number of dice rolled and of the ring the dart hit: odd (blue) vs. even (pink). Error bars indicate within-subject +/- 1 standard error of the mean.

1. **Additional Materials for Exp-2** **(description-condition)**

Tables indicating players' kicking style and success rates as shown to the participants in this condition:

"Purple player style and success rates, based on previous two seasons":

| **Kick style:** | Around the wall |
| --- | --- |
| **Success rates:** | 60% for a goal |

"Red player style and success rates as collected from previous two seasons":

| **Kick style:** | Through the wall |
| --- | --- |
| **Success rates:** | 30% for a goal |

"Blue player style and success rates as collected from previous two seasons":

| **Kick style:** | Through the wall |
| --- | --- |
| **Success rates:** | 60% for a goal |

"Cyan player style and success rates as collected from previous two seasons":

| **Kick style:** | Around the wall |
| --- | --- |
| **Success rates:** | 30% for a goal |

1. **Materials in Experiment 3 and post-experimental test in Experiment 4**

English translation of the text presented to participants (in Hebrew) in experiment-3.


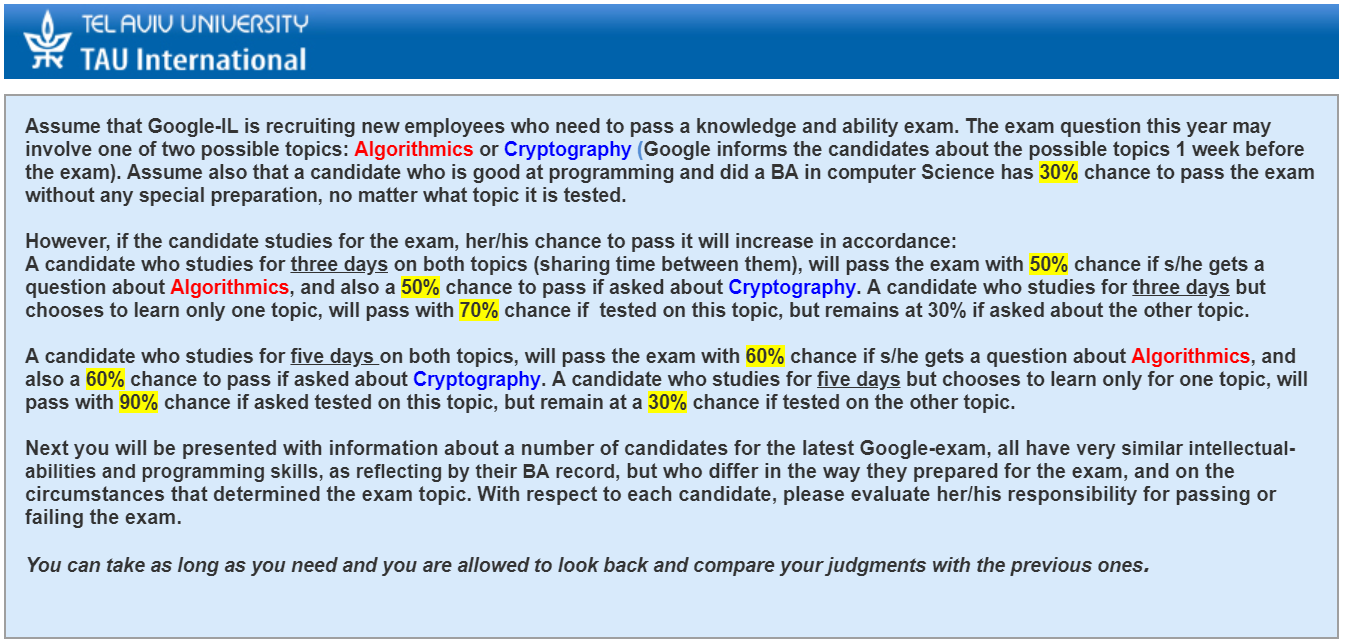


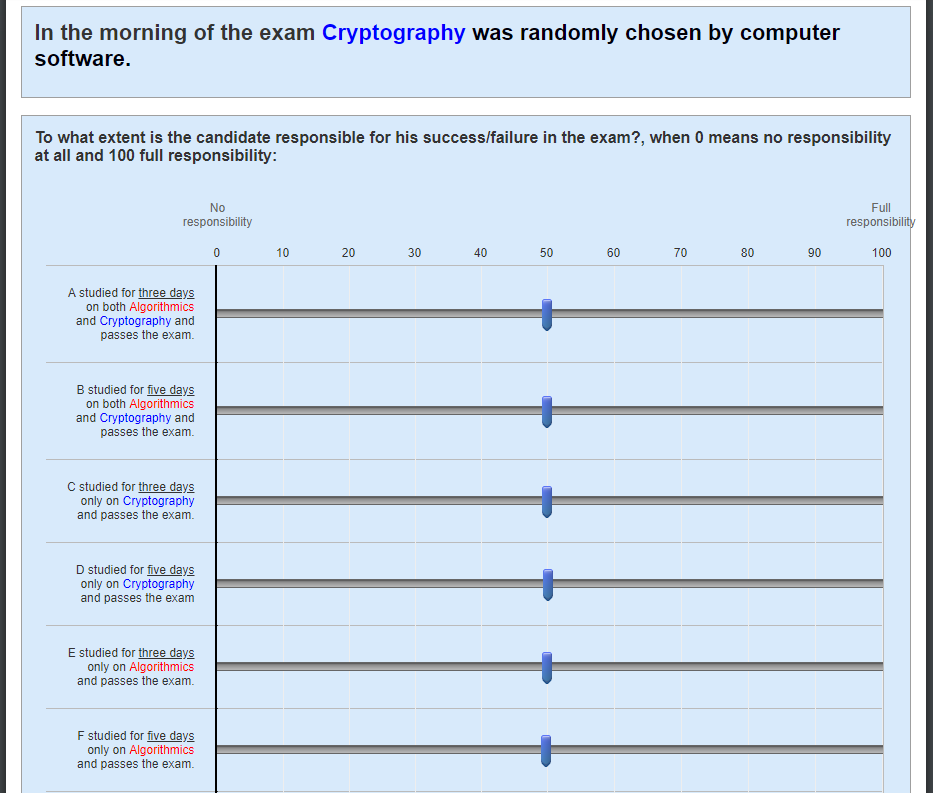


*C.1 Memory post-test for success rate in experiment 4*


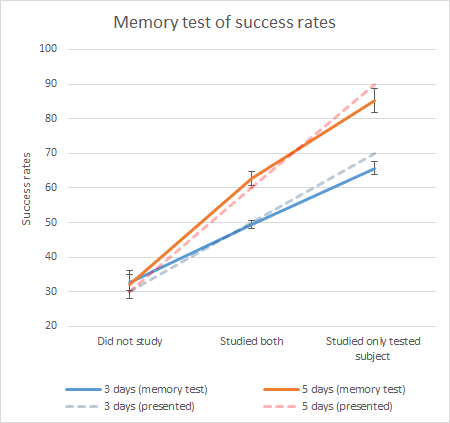


Figure S2. Results of the post-experimental memory quiz for the success rate of the exam candidates in all cases of Exp-4. Dotted lines are nominal values and solid lines are the average report (memory). Error bars correspond to SE of the Mean.
